# The Official Conditions for Obtaining Grants

**Published:** 1915-08-14

**Authors:** 


### MOTHERCRAFT AND CHILD-WELFARE CENTRES.

### The Official Conditions for Obtaining Grants.

Economy in all directions, except the obvious one of tobacco-smoking, is now being vigorously preached, and there are signs that even public bodies are earnestly considering effective retrenchment. Statesmanlike qualities are needed for the difficult task of discriminating between the many schemes which local authorities now have in working order. At the present moment it should be easy to decide that one class of municipal or local enterprise is deserving not only of continued support, but of steady amplification. In that category all thinking men and women will place those measures devoted to the dissemination of a knowledge of what is now called mothercraft, and to the promotion of the welfare of children by educating their parents in the medical and hygienic aspects of child welfare. Under this heading are included Schools for Mothers, Baby Clinics, and Maternity Centres.

Such measures are beginning to receive substantial encouragement from the Board of Education and the Local Government Board. These two authorities are now co-operating, and, in future, grants from either of these bodies will be co-ordinated and regulated more precisely than hitherto. A recent circular (No. 906), issued jointly by these authorities, refers to the modifications, which experience has shown to be desirable, of the arrangements under which grants are paid by the Board of Education and the Local Government Board respectively in aid of the work of institutions or agencies known as Schools for Mothers, Baby Clinics and Dispensaries, or Maternity Centres. Since these aim at promoting the health of mothers and little children not registered in day nurseries, nursery schools, or public elementary schools, the Board recognises them as providing a hitherto missing link in the chain of educational institutions.

The above-mentioned circular (No. 906) refers to grants which will be made in respect of work done in the financial year commencing April 1, 1915, and, as far as possible, it will be arranged that all State aid to a single institution or agency shall be given by one department only, regard being had to the predominant character of the institution.

Since Circular 906 was issued, regulations have been published both by the Local Government Board and by the Board of Education. The grants of the former authority will be made under the general heading of grants for maternity centres. In respect of the following services the Local Government Board will pay grants during the year ending March 31, 1916:—The salaries and expenses of inspectors of midwives and of health visitors; the provision of a midwife or doctor for the aid in confinement of necessitous women; the expenses of a maternity centre, which is defined as an institution providing any or all of the following activities: medical supervision and advice for expectant and nursing mothers, and for infants and little children, and medical treatment for cases needing it. Both sets of regulations come into force as from April 1, 1915.

The grants will be assessed on the basis of the expenditure incurred on these services, and they amount to one half of this expenditure if the services are adequate and sufficient. Voluntary agencies may receive grants if their work is approved by the Board, and is co-ordinated as far as practicable with the public health work of the local sanitary authority and with the school medical service of the local education authority. The premises and work must be subject to inspection, and satisfactory records of the work done must be kept.

The Board of Education will make grants to schools for mothers "in respect of the provision made for promoting the care, training, and physical welfare of infants and young children." The regulations define a school for mothers as primarily an educational institution, providing training and instruction for the mother in the care and management of infants and little children. The imparting of such instruction may include systematic classes, home visiting, and infant consultations.

### An Official Warning.

It is expressly stated in the regulations that the provision of specific medical and surgical advice or treatment (if any) should be only incidental. This warning is doubtless intended to discourage any tendency for these schools for mothers to become miniature out-patient departments rather than centres for instruction. No grants will be paid by the Board of Education to institutions which do not provide collective instruction by means of systematic classes, or to infant consultations which are not provided for women attending a school for mothers. Agencies provided by a Sanitary Authority or a County Council, and any work of home visiting in connection with a school for mothers which is undertaken by a Sanitary Authority or County Council, will not receive grants, such grants, presumably, being payable by the Local Government Board. But the fact that a school for mothers receives a grant from a Sanitary Authority will not disqualify it from receiving a grant from the Board of Education.

The amount of the grants may equal one-half the approved expenditure, as in the case of those given by the Local Government Board, and similar conditions apply. Regard will be paid to the provision made for co-ordinating the work of the institution with that of similar institutions in the same district, with maternity centres and baby clinics providing medical advice or treatment, and with the School Medical Service on the one hand, and the Sanitary Authority on the other. It is further mentioned in these regulations that the institution must be conducted by a responsible body of managers, and a correspondent must be appointed on behalf of the managers. The institution must not be conducted for private profit or farmed out to any member of the staff. "D.P.H."

